# Bio‐inspired hierarchical bamboo‐based air filters for efficient removal of particulate matter and toxic gases

**DOI:** 10.1002/EXP.20240012

**Published:** 2024-06-10

**Authors:** Qi Gao, Jian Gan, Pixiang Wang, Yuxiang Huang, Daihui Zhang, Wenji Yu

**Affiliations:** ^1^ Research Institute of Wood Industry Chinese Academy of Forestry Beijing China; ^2^ Jiangsu Co‐Innovation Center of Efficient Processing and Utilization of Forest Resources Nanjing Forestry University Nanjing China; ^3^ Institute of Chemical Industry of Forest Products Chinese Academy of Forestry Nanjing China; ^4^ Center for Materials and Manufacturing Sciences Department of Chemistry and Physics Troy University Troy USA

**Keywords:** bamboo cellulose fibers, formaldehyde adsorption, hierarchical air filters, metal−organic frameworks, particulate matter removal

## Abstract

Air pollution is caused by the perilous accumulation of particulate matter (PM) and harmful gas molecules of different sizes. There is an urgent need to develop highly efficient air filtration systems capable of removing particles with a wide size distribution. However, the efficiency of current air filters is compromised by controlling their hierarchical pore size. Inspired by the graded filtration mechanisms in the human respiratory system, microporous ZIF‐67 is in situ synthesized on a 3D interconnected network of bamboo cellulose fibers (BCFs) to fabricate a multiscale porous filter with a comprehensive pore size distribution. The macropores between the BCFs, mesopores formed by the BCF microfibers, and micropores within the ZIF‐67 synergistically facilitate the removal of particulates of different sizes. The filtration capabilities of PM2.5 and PM0.3 could reach 99.3% and 98.6%, respectively, whereas the adsorption of formaldehyde is 88.7% within 30 min. In addition, the filter exhibits excellent antibacterial properties (99.9%), biodegradability (80.1% degradation after 14 days), thermal stability, and skin‐friendly properties (0 irritation). This study may inspire the research of using natural features of renewable resources to design high‐performance air‐filtration materials for various applications.

## Graphical abstract

Microporous ZIF‐67 is synthesized in situ on the 3D interconnected network of bamboo cellulose fibers to produce a multiscale porous filter with a comprehensive pore size distribution. The porous filter has an excellent filtration effect on the particles particulate matter (PM, including PM10, PM2.5 and PM0.3) and harmful gas molecules in the air.

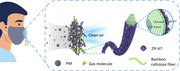

## 1 INTRODUCTION

Access to clean air is a fundamental necessity for human life.^[^ ^1^, ^2^ ^]^ However, rapid economic growth, globalization, and increasing energy demands have generated substantial quantities of harmful waste, causing severe air pollution.^[^ ^3^, ^4^, ^5^ ^]^ The detrimental effects of air pollution can deteriorate the social economy and human health. According to the data from the World Health Organization, air pollution is linked to cardiovascular diseases, respiratory illnesses, and cancer, resulting in approximately seven million premature deaths annually.^[^ ^6^, ^7^, ^8^, ^9^ ^]^Air pollutants include particulate matter (PM), such as dust, smoke, and fog, and gaseous substances, such as formaldehyde and sulfur dioxide.^[^ ^10^, ^11^ ^]^ PM can be divided into different levels according to the size of particulate matter, such as PM0.3, PM2.5, PM10 etc.^[^ ^12^ ^]^ PM2.5 can penetrate the human bronchi and lungs, triggering inflammation and disease in the body.^[^ ^13^, ^14^ ^]^ PM0.3 with a smaller particle size can even enter the blood system and produce toxic effects on the cardiovascular system.^[^ ^15^, ^16^ ^]^ Therefore, there is an urgent need to develop high efficiency air filtration systems that can capture different particle sizes of PM and a variety of chemical gases.

Traditional commercial air filtration materials are mainly made of polypropylene (PP), polyethylene (PE), polyurethane (PU) and glass fiber.^[^ ^17^, ^18^, ^19^ ^]^ They are less efficient in removing PM0.3 due to the lack of active functional groups.^[^ ^20^ ^]^ Recently, air filters based on electrospinning nanofibers, such as polyacrylonitrile (PAN), polyvinylidene fluoride (PVDF), polyvinyl alcohol (PVA), and polyvinyl chloride (PVC), have attracted much attention and become one of the main strategies in preparing air filters.^[^ ^21^, ^22^, ^23^, ^24^, ^25^, ^26^, ^27^ ^]^ These fibers exhibit a significantly improved filtration performance due to the significantly high specific surface area. However, most of the above raw materials are non‐renewable and difficult to degrade, which will potentially produce secondary pollution and has a huge impact on the environment.^[^ ^28^, ^29^ ^]^ In order to enhance environmental protection, air filters produced from chitosan, zein, filamin, soybean protein, gelatin and other raw materials have been reported.^[^ ^30^, ^31^, ^32^ ^]^ However, the pore size of these nanofiber filters is usually very small. As a result, a high‐pressure drop is almost inevitable. To reduce the pressure drop, the researchers have created ribbon fibers, extremely fine fibers, and thin filters,^[^ ^33^, ^34^, ^35^ ^]^ but the manufacturing process is complex and expensive. Owing to the single nanostructure, it is difficult to balance the various properties in the air filter, so its practical application is severely limited. Therefore, it is very urgent to balance the filtration performance, pressure drop and environmental protection with a reasonable cost. Although the hierarchical structure air filters combining nanoscale and micrometer scale have recently emerged,^[^ ^36^ ^]^ there is still a pressing need to develop highly efficient air filtration systems with comprehensive and multiscale pore structures. Metal–organic frameworks (MOFs) are porous framework compounds composed of metal ions and organic ligands with abundant micropores, large specific surface areas, and tunable surface charges.^[^ ^37^, ^38^, ^39^ ^]^ As nanoscale porous materials, MOFs can capture fine particulates and toxic gas molecules via electrostatic adsorption, showing promising applications for air filtration. Notably, ZIF‐67, a significant subclass of MOFs, exhibits exceptional characteristics, including a remarkably large specific surface area and high chemical stability, as evidenced by numerous studies.^[^ ^40^, ^41^, ^42^ ^]^ However, the poor flexibility and mechanical properties of ZIF‐67 powder restrict its applications.^[^ ^43^ ^]^ Therefore, it is necessary to find a suitable matrix for ZIF‐67 powder to overcome its limitations and maximize its advantages. Various filtration substrates have been studied, such as chitin aerogels, polypropylene nonwovens, polycarbonate fiber membranes, and electrospun nylon membranes.^[^ ^44^, ^45^, ^46^, ^47^, ^48^ ^]^ However, the pore sizes of these materials are relatively single. It is difficult to fully filter the particles of all sizes and gas molecules. Therefore, the development of filter materials with abundant pores remains a significant challenge. Bamboo is one of the most available biomass materials on Earth, offering numerous advantages such as eco‐friendliness, a low carbon footprint, and cost‐effectiveness.^[^ ^49^ ^]^ Bamboo cellulose fibers (BCFs) can be extracted from natural bamboo.^[^ ^50^ ^]^ As a micrometer‐scale substrate, BCFs's unique hollow structures comprising mesopores and macropores create intricate pathways for particulate crossing, thereby enhancing the possibility of effective collisions between the filter and the particulates. At the same time, BCFs offer abundant binding sites, low airflow resistance, and excellent mechanical strength, making them an excellent base material for air filtration.^[^ ^51^, ^52^, ^53^ ^]^ In addition, compared to other commercial petroleum‐based materials,^[^ ^54^, ^55^ ^]^ BCFs are safe and biodegradable, mitigating the risk of secondary pollution. Unfortunately, to the best of our knowledge, BCFs have not been investigated as filtration substrates for air purification.^[^ ^56^, ^57^ ^]^

Inspired by graded filtration in human respiration, the pore structure in BCFs was combined with the micropores of ZIF‐67 to design a hierarchical air filter (ZIF‐67@BCFs) to filter pollutants of various sizes. Importantly, the entire preparation process is environmentally friendly and straightforward without using organic solvents, such as *N*,*N*‐dimethylformamide, which is commonly employed in MOFs composite materials. The ZIF‐67@BCFs showed substantial specific surface area (442 m^2^ g^−1^), enabling outstanding removal efficiencies for PM_10_ (99.9%), PM_2.5_ (99.3%), PM_0.3_ (98.6%), total volatile organic compounds (91.1% within 30 min), and formaldehyde (88.7% within 30 min). Furthermore, these materials exhibited excellent washability, reusability, thermal stability, biodegradability (80% degradation in 14 days), antibacterial properties (99.9%), and allergy prevention characteristics (zero stimulus). This study offers a novel strategy to develop sustainable, multifunctional, and environmentally friendly air‐filtration materials.

## 2 RESULTS AND DISCUSSION

**FIGURE 1:**
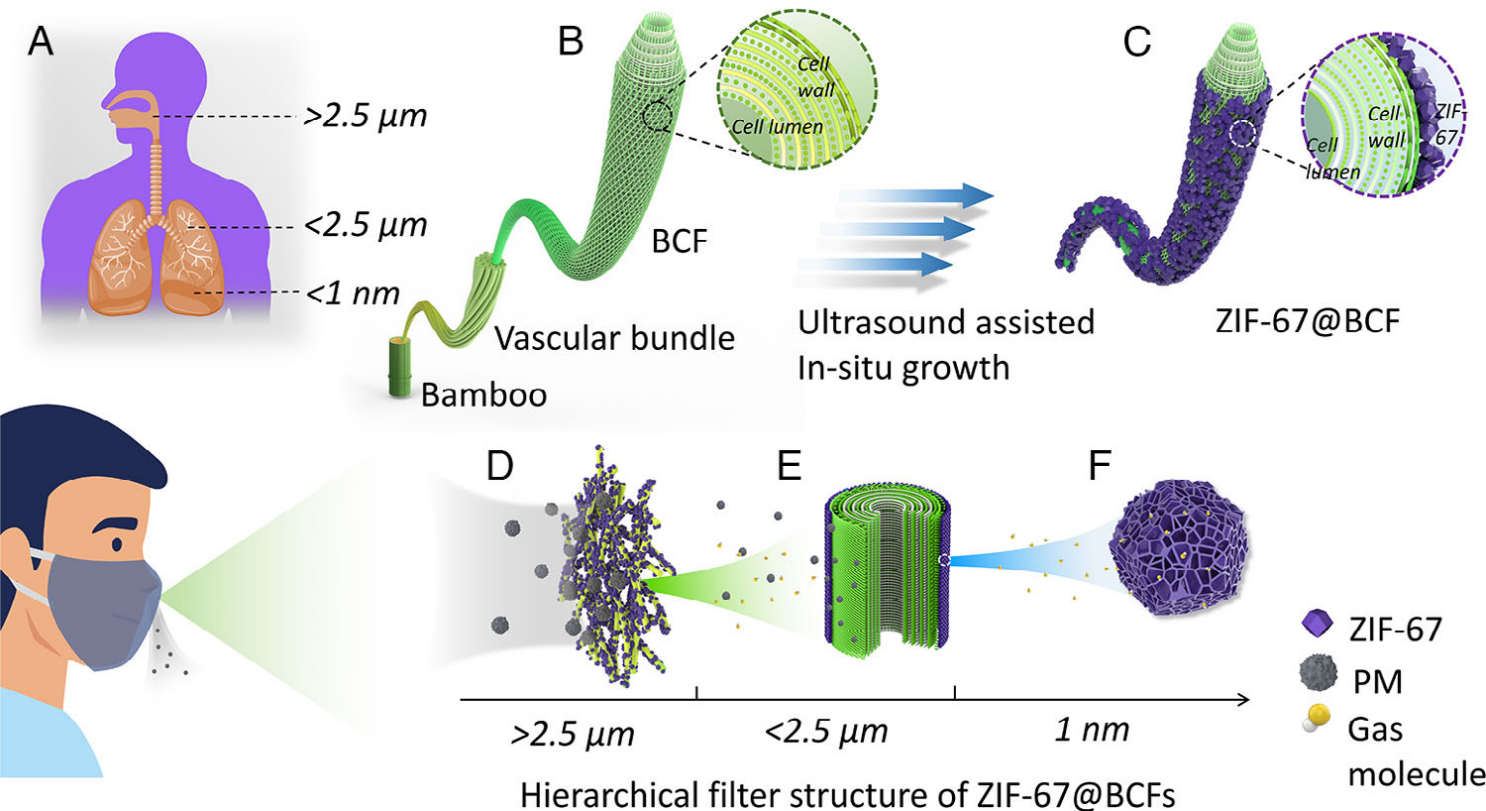
(A) Schematic diagram of the filtration process of the human respiration system. Synthesis process from (B) BCFs to (C)ZIF‐67@BCFs. Hierarchical filtration process of ZIF‐67@BCFs for (D) large diameter particulates, (E) small diameter particulates, and (F) gas molecules.

Figure 1A shows the filtration process of the human respiration system, which can self‐defend and filter solid particulates and harmful gas molecules from the air. The entire filtration process is divided into three stages according to the different sizes of the PM. Primary filtration removes crude particles greater than 2.5 μm through the adhesion of nasal and tracheal folds and villi fibers. Particulates less than 2.5 μm are eliminated by secondary filtration through the contact adsorption of bronchus and alveoli. The third stage of filtration involves molecular filtration of particulates and harmful gases with diameters of less than 1 nm through blood vessels. Eventually, the clean air is eventually transported into the blood circulation system. Inspired by pulmonary respiration filtration, a hierarchical air‐filtration material was designed using natural BCFs as the skeleton in combination with MOF particles. Specifically, ZIF‐67 with abundant micropores was uniformly grown on BCFs through an ultrasonic process (Figure 1B) to construct a high‐efficiency air filter with a full aperture distribution (ZIF‐67@BCFs, Figure 1C). As a result, the particulates with a diameter larger than 2.5 μm were physically blocked through the three‐dimensional network generated by ZIF‐67@BCFs (Figure 1D). Secondary filtration involves the electrostatic adsorption of particulates smaller than 2.5 μm to the natural pore structure of BCFs (Figure 1E), while the adsorption of particulates with a diameter less than 1 nm and harmful gases can be accomplished through the rich micropores in ZIF‐67 (Figure 1F). The ZIF‐67@BCFs with multiscale pore structures can completely and efficiently remove PM and harmful gases, thereby serving as a highly promising filtration system.

**FIGURE 2:**
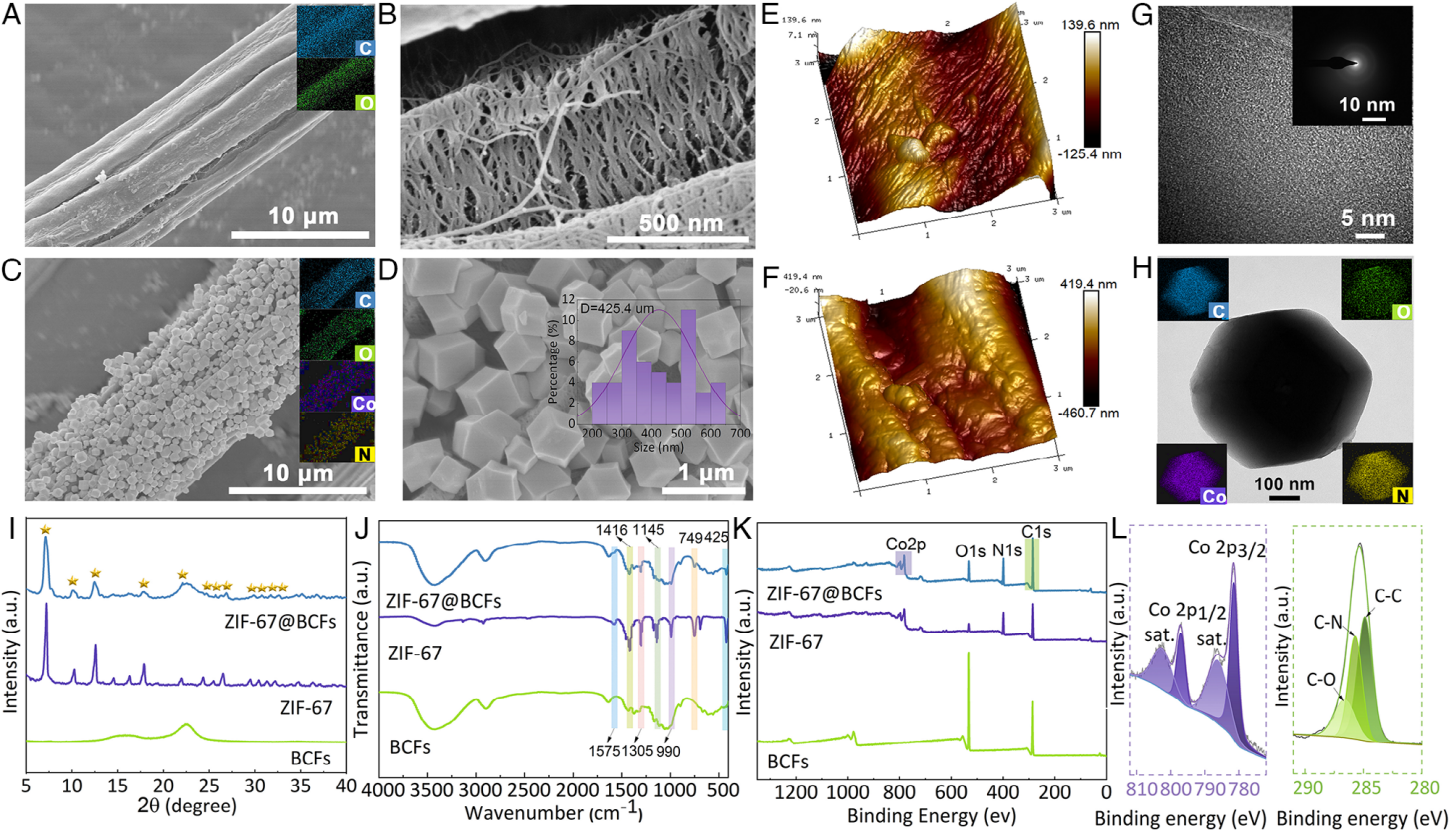
SEM images of BCFs (A, B), ZIF‐67@BCFs (C, D). AFM topography images of BCFs (E), ZIF‐67@BCFs (F). (G) HRTEM image of ZIF‐67@BCFs; inset: corresponding SAED image. (H) TEM image and EDX mapping of ZIF‐67@BCFs. (I) XRD patterns, (J) FTIR spectra, (K) XPS spectra of ZIF‐67, BCFs, and ZIF‐67@BCFs. (L) Deconvolution of C and Co peaks.

The morphologies of the BCFs and ZIF‐67@BCFs were studied using scanning electron microscopy (SEM) and energy dispersive X‐ray analyzer (EDX). Figure 2A shows the smooth and defect‐free surface of BCFs with minimal pits. The BCFs formed a 3D network of microfibers with a porous structure (Figure 2B) as the multilayers of cellulose fibrils were embedded in the hemicellulose and lignin matrices. After matrix removal, the BCFs developed an interconnected porous structure. Simultaneously, supercritical drying reduced the capillary pressure at the vapor‐liquid interface and limited the microfiber aggregation, retaining more pores in BCFs.^[^ ^50^ ^]^ The porous and interconnected 3D network of the BCFs served as a physical anchor for ZIF‐67 nucleation, enhancing the effective contact area between ZIF‐67 and the cellulose molecular chain. As shown in Figure S1A,B, Supporting Information, the untreated original bamboo fibers (BFs) had limited pore structures, insufficient for the nucleation of ZIF‐67 particles. As a result, the loading rate of ZIF‐67 on BFs (5%) was considerably lower than that on BCFs, and the growth of ZIF‐67 was uneven (Figure S1C,D, Supporting Information). In addition, ZIF‐67 loading on naturally air‐dried bamboo cellulose fibers (BCFs‐1) was not ideal. Compared to the supercritical dried BCFs, the ZIF‐67 load rate and specific surface area of BCFs‐1 were reduced by 37% and 88%, respectively (Figure S1E,F, Supporting Information). It revealed that the porous BCFs prepared in this study offered a significant advantage for in situ ZIF‐67 growth. As shown in Figure 2C, after in situ ZIF‐67 growth, the BCFs were uniformly covered by dense ZIF‐67 nanoparticles. Additionally, the effect of solvent on the morphology and distribution of ZIF‐67 in the BCFs was studied. When water was used, lamellar ZIF‐67 was formed when the molar ratios of cobalt nitrate hexahydrate and 2‐methylimidazole molar were 1:8 and 1:15. At a molar ratio of 1:30, ZIF‐67 nanoparticles with irregular shapes and uneven sizes were obtained (Figure S2, Supporting Information) due to the faster growth kinetics in water.^[^ ^58^ ^]^ In the case of methanol, the size of ZIF‐67 nanoparticles was non‐uniform at room temperature (Figure S3, Supporting Information). However, under ultrasonication, the morphology of ZIF‐67 became uniform and regular, with an average particle diameter of 425.4 nm and an even distribution along the fibers (Figure 2D). Therefore, methanol was employed as the solvent to prepare ZIF‐67@BCFs with the assistance of ultrasonication. The EDX diagrams of C, O, Co, and N further verified the uniform ZIF‐67 loading on the BCFs surfaces. Atomic force microscopy (AFM) was used to characterize the surface roughness of the BCFs and ZIF‐67@BCFs. The arithmetic average roughness (*R*_a_) of the raw BCFs was 53.6 nm (Figure 2E). After the deposition of the ZIF‐67 particles, the *R*_a_ value of the ZIF‐67@BCFs increased to 148.1 nm (Figure 2F). This illustrated the micro/nanoscale roughness formed on the surfaces of the BCFs, confirming the successful loading of ZIF‐67. The transmission electron microscopy (TEM) image revealed that the Co atoms were uniformly distributed within the ZIF‐6@BCFs without particle aggregation (Figure 2G). The selected region electron diffraction (SAED) pattern exhibited no dot or ring patterns, implying the amorphous states of Co species.^[^ ^59^ ^]^ The TEM images and EDX plots suggested the well‐dispersion of Co, N, O, and C within ZIF‐67@BCFs (Figure 2H).

X‐ray diffraction (XRD) profiles were used to analyze the phase structures of ZIF‐67, BCFs, and ZIF‐67@BCFs (Figure 2I). BCFs showed characteristic peaks at 2*θ* = 22.3 (200), 16.4 (110), and 15.5 (110), respectively. Compared with BCFs, ZIF‐67@BCF exhibited distinct new diffraction peaks at 7.4°, 10.4°, 12.7°, 18.1°, 22.1°, 24.6°, 25.7°, 26.8°, 29.6°, 30.7°, 31.7°, and 32.4°, corresponding to (011), (002), (112), (222), (114), (233), (224), (134), (034), (334), (244), and (235) planes of ZIF‐67 crystals, respectively.^[^ ^60^ ^]^ The surface chemical structure of ZIF‐67, BCFs, and ZIF‐67@BCFs were further characterized using Fourier infrared spectroscopy (FTIR) and X‐ray photoelectron spectroscopy (XPS). As shown in Figure 2J, the absence of the characteristic peaks at 1512 cm^−1^ (C═C of the aromatic nucleus) and 1736 cm^−1^ (C═O group) in the BCFs indicated the complete removal of lignin and hemicellulose. The broad band at 3000–3800 cm^−1^ was attributed to the mixing of intermolecular (─O6H···O3H), intramolecular (O2H···O5, O2H···O6), free (O2H, O6H) hydrogen bonds, and hydrogen bonds between water molecules and cellulose chains.^[^ ^50^ ^]^ After in situ ZIF‐67 growth on the BCFs, new bands related to ZIF‐67 were observed. The peaks at 1575 and 1416 cm^−1^ were caused by the stretching vibrations of the imidazole ring C─N.^[^ ^58^ ^]^ The peaks at 1145 and 990 cm^−1^ were attributed to the in‐plane bending vibration of the imidazole group, and the peak at 749 cm^−1^ corresponded to the out‐of‐plane bending vibration of the imidazole group.^[^ ^61^ ^]^ The C─H vibration and bending vibrations of the Co─N group of ZIF‐67 were observed at 1305 and 425 cm^−1^, respectively.^[^ ^62^ ^]^ In addition, the XPS spectra showed two new peaks of BCFs at 778.6 (Co 2p) and 400.9 eV (N 1s) after modification with ZIF‐67 (Figure 2K). The peak splitting results for Co showed that the binding energies of Co 2p3/2 and Co 2p1/2 were at 781.4 and 796.9 eV with two satellite peaks at 786.1 and 802.5 eV, respectively, revealing the presence of Co^2+^ cations in ZIF‐67@BCFs (Figure 2L). The peak deconvolution of C showed a C─N peak (285.9 eV), indicating the presence of an imidazole ring. These analyses further confirm the in situ growth of ZIF‐67 in the BCFs.

**FIGURE 3:**
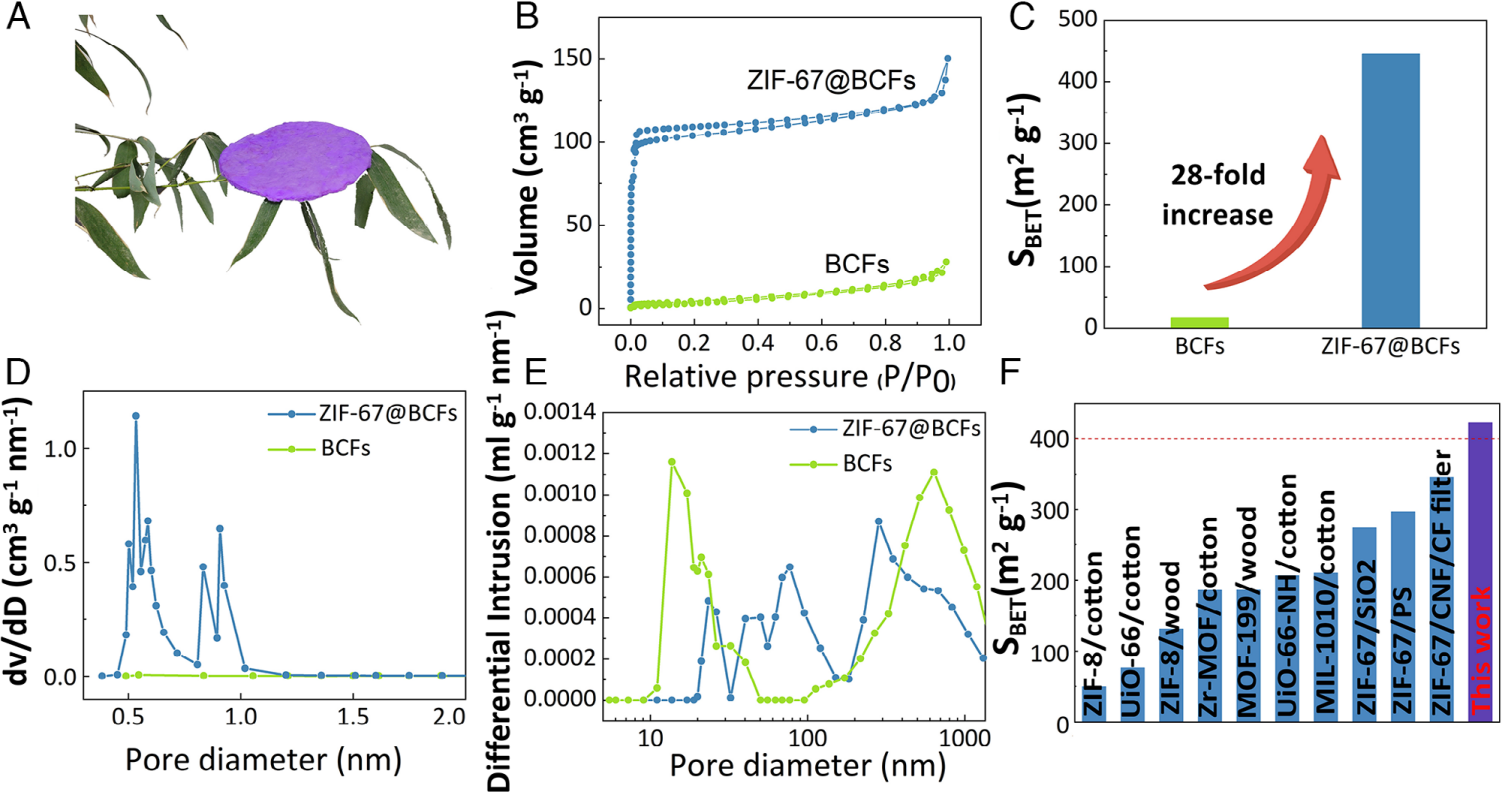
(A) Photo of a circular ZIF‐67@BCFs. (B) N_2_ adsorption capacity, and (C) specific surface areas of BCFs and ZIF‐67@BCFs. (D, E) pore distributions of BCFs and ZIF‐67@BCFs. (F) BET surface areas (*S*_BET_) of ZIF‐67@BCFs and other air filtration materials.

As shown in Figure 3A, ZIF‐67@BCFs of various shapes were fabricated, including circles and squares. The circular ZIF‐67@BCFs had a diameter of 120 mm, a thickness of 1 mm, and a mass of 1.25 g. This lightweight material is highly suitable for air‐filtration applications. The adsorption performance of ZIF‐67@BCFs and ZIF‐67 was investigated by Brunauer–Emmett–Teller (BET) experiments. Both crystals showed type I adsorption isotherms (Figure 3B). Furthermore, ZIF‐67@BCFs demonstrated a significantly higher N_2_ adsorption capacity than BCFs, confirming the enhanced porosity after ZIF‐67 deposition. The BET specific surface area was 16.3 m^2^ g^−1^ for BCFs and increased to 445.2 m^2^ g^−1^ for ZIF‐67@BCFs (Figure 3C), suggesting that the porous and interconnected 3D network of BCFs enabled the adequate growth of ZIF‐67 particles.

The pore size and distribution of air‐filtration materials are also important for filtration efficiency. The BCFs possess natural macropores and mesopores, which are beneficial for ZIF‐67 loading. After in situ ZIF‐67 growth onto BCFs, the mean pore size of BCFs decreased significantly from 10.45 to 1.96 nm (Figure 3D,E) because of the micropores in ZIF‐67 (average pore size of 1.6 nm, Figure S5, Supporting Information). However, the total pore volume increased five‐fold from 0.0425 to 0.2171 mL g^−1^. Compared to recently reported air filtration materials, the specific surface area of the ZIF‐67@BCFs in this study was exceptionally high (Figure 3F). Therefore, the combination of multiscale natural bamboo structures and ZIF‐67 nanoparticles is promising for preparing air‐filtration materials with enhanced porosity and adsorption performance.

**FIGURE 4:**
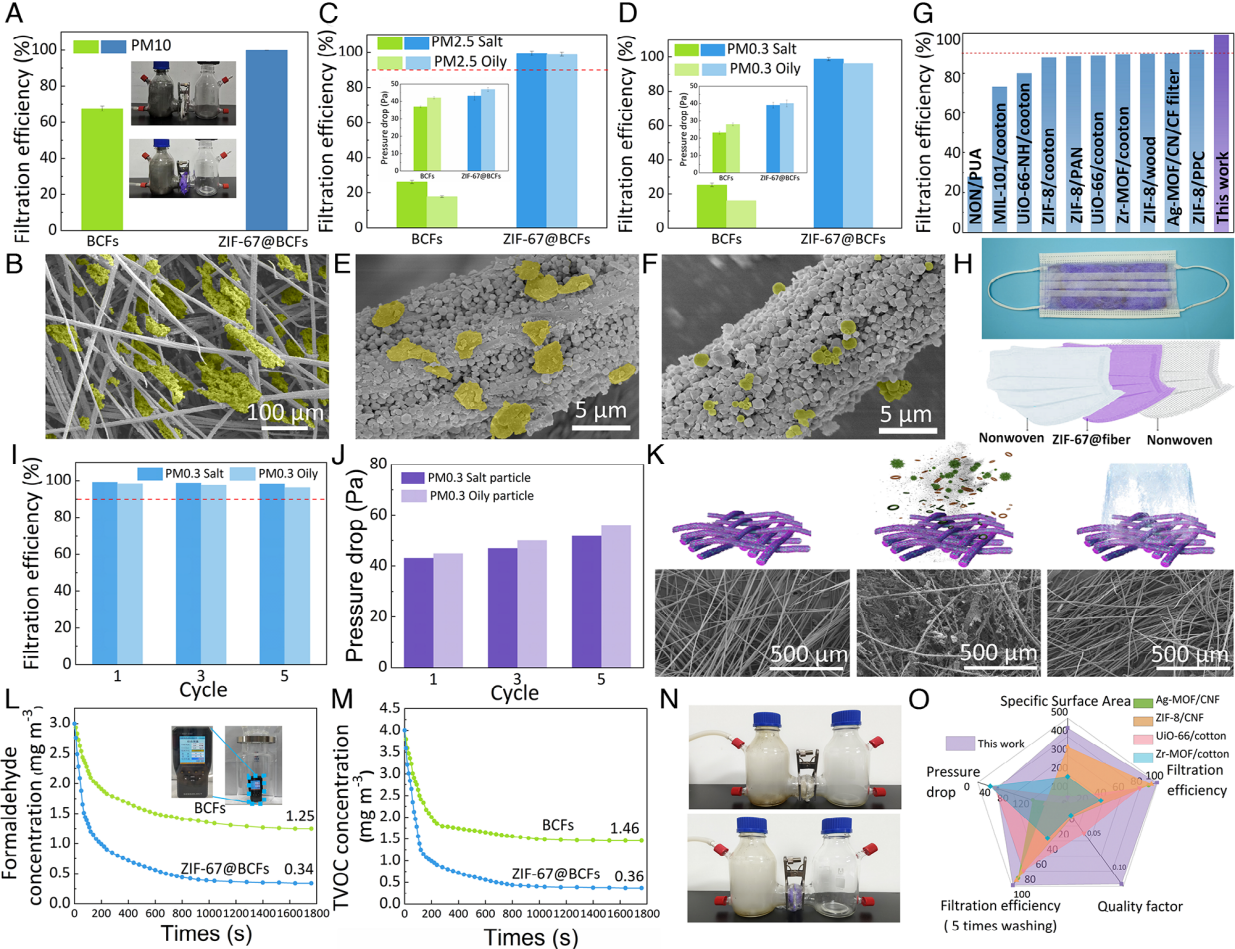
Filtration performance of BCFs and ZIF‐67@BCFs for (A) PM_10_, (B) PM_2.5_, and (C) PM_0.3_. (D) Filtration performance of ZIF‐67@BCFs and the other air filtration materials. SEM images of ZIF‐67@BCFs after filtering (E) PM_10_, (F) PM_2.5_, and (G) PM_0.3_. (H) ZIF‐67@BCFs homemade mask. (I) Filtration efficiency, (J) Pressure drop, and (K) Mechanism properties of ZIF‐67@BCFs after washing. Adsorption properties of ZIF‐67@BCFs for (L) formaldehyde and (M) TVOC. (N) Filtration performance of ZIF‐67@BCFs for smoke. (O) A comprehensive comparison between ZIF‐67@BCFs and other air filtration materials.

Air pollutants are primarily composed of particulates, volatile organic compounds (VOCs), and formaldehyde. Prolonged inhalation of these pollutants can induce various inflammatory responses in the human respiratory, digestive, and circulatory systems, potentially leading to severe outcomes like leukemia.^[^ ^63^ ^]^ Therefore, there is a significant interest in the development of safe and effective air‐filtration materials in academia and industry. To assess the practical filtration performance of the ZIF‐67@BCFs, gas filtration experiments were conducted using PM_10_ (large‐diameter particulates), PM_2.5_ and PM_0.3_ (small‐diameter particulates), as well as harmful gas molecules (VOCs and formaldehyde). Large‐particle dust pollution was simulated by a customized instrument, and a particle counter was used to calculate the filtration efficiency (Videos S1 and S2 and Figure S6A, Supporting Information). As shown in Figure 4A, the BCFs exhibited a filtration efficiency of 67.5% owing to their natural porous structures. Interestingly, the filtration efficiency of ZIF‐67@BCFs increased sharply to 99.9%. SEM analysis showed that the PM_10_ particulates were intercepted by the 3D network structure (Figure 4B), suggesting that the dense networks in ZIF‐67 might prompt electrostatic interactions, thereby increasing the filtration efficiency.

The filtration efficiencies of the BCFs for PM_2.5_ (26.3% for saline particulates and 17.8% for oily particulates) were significantly lower than those for PM_10_, indicating that particulates with a diameter of less than 2.5 μm could penetrate the macropores in the 3D network structure of natural bamboo fibers (Figure 4C). In contrast, ZIF‐67@BCFs achieved PM_2.5_ filtration efficiencies of 99.3% for saline particulates and 98.9% for oily particulates. The ZIF‐67@BCFs attained a quality factor of up to 0.116 (Figure S6B, Supporting Information). A similar trend was observed for PM_0.3_ (Figure 4D), with a filtration efficiency of 98.6% and a quality factor of 0.102 (Figure S6C, Supporting Information). From the SEM images, PM_2.5_ and PM_0.3_ were mainly adsorbed on the ZIF‐67 surface and the mesopores of the BCFs (Figure 4E,F). The high filtration efficiency of ZIF‐67@BCFs could be attributed to the following reasons. (1) The unique 3D network structure of the BCFs accommodated abundant ZIF‐67 nanoparticles, significantly enhancing the porosity and specific surface area of the filtering material. (2) The imbalanced metal ions and defects in ZIF‐67 polarized the PM surface, improving the electrostatic interactions between ZIF‐67 and the PM (Figure S6D, Supporting Information).^[^ ^63^ ^]^ (3) The 3D network structure of the BCFs mimicked the hierarchical structure of the lung and facilitated gradient filtration, capturing more particulates and creating a complex and tortuous channel for PM particulates, thereby increasing the frequency of effective collisions between the filters and particulates. Therefore, ZIF‐67@BCFs air filters demonstrate great potential for removing PMs of various sizes.

A comparison of the PM_2.5_ filtration performance revealed that ZIF‐67@BCFs exhibited a higher efficiency (99.3%, Figure 4G) than MIL‐100/cotton (73.2%), UiO‐66‐NH‐SO3H@cotton (80.0%), ZIF‐8@cotton (87.7%), UiO‐66@cotton (88.4%), ZIF‐8/PAN MO filter (88.3%), Ag‐MOFs@CNF@CF filter (90.0%) and Zr‐MOF‐NO2@cotton (89.5%). In addition, the applicability of ZIF‐67@BCFs was verified by fabricating a homemade mask with extracting and drying the ZIF‐67@BCFs as the middle layer and the nonwovens as the inner and outer layer (Figure 4H). Nonwovens have very little effect on the porosity and filter pressure drop, so the pore distribution and characteristics of the mask components are largely consistent with those of ZIF‐67@BCFs. To evaluate the reliability of ZIF‐67@BCFs, the thin ZIF‐67@BCFs were subjected to five wash cycles and then tested for filtration performance and pressure drop. As shown in Figure 4I, ZIF‐67@BCFs maintained high filtration performance after five washes (98%). A high filtration efficiency of 97% was maintained after 15 cycles (Figure S6E, Supporting Information). At the same time, the pressure drop showed no obvious increase (Figure 4J), implying that most of the trapped particulates in the pores could be simply removed after washing (Figure 4K). Therefore, the ZIF‐67@BCFs exhibited excellent reusability.

Total volatile organic compounds (*T*_VOC_) comprise a variety of volatile organic substances, such as benzene, toluene, and ethylbenzene. They originate from indoor and outdoor pollution sources and pose risks to human health and the environment. The *T*_VOC_ adsorption properties of the BCFs and ZIF‐67@BCFs were tested (Figure S6G, Supporting Information). The initial mass concentration of the *T*_VOC_ in the sealed device was maintained at 4 mg m^−3^. Upon introducing the ZIF‐67@BCFs into the glass‐sealed container, the *T*_VOC_ mass concentration decreased rapidly (Figure 4L). Within 30 min, the *T*_VOC_ concentration decreased to 0.36 mg m^−3^ (removing 91.0% of the VOC) and reached a plateau. Moreover, formaldehyde adsorption of ZIF‐67@BCFs followed a similar trend (Figure 4M). The concentration rapidly decreased within 5 min and stabilized at 0.36 mg m^−3^ after 30 min. Compared to the BCFs, the ZIF‐67@BCFs exhibited significantly improved adsorption capacities for *T*_VOC_ and formaldehyde, making them highly suitable for indoor and outdoor air filtration. In addition, the formaldehyde adsorption capacity of ZIF‐67@BCFs also has significant advantages over activated carbon (Figure S6H, Supporting Information).

Currently, incense is widely used to improve indoor air quality. However, research has shown that incense combustion emits various particulates and harmful gas molecules.^[^ ^64^ ^]^ Thus, incense was used to create a polluting condition containing large particulates, fine particulates, and harmful gas molecules in the air (Videos S3 and S4, Supporting Information). When the BCFs were placed at the junction of the two glass bottles for filtration, the smoke was observed in the glass bottle on the right (Figure 4N). However, when ZIF‐67@BCFs were used as the filter material, smoke was completely adsorbed with no gases escaping to the bottle on the right. This demonstrated that ZIF‐67@BCFSs effectively blocked the flow of smoke and filtered harmful substances.

The overall filtering properties of ZIF‐67@BCFs were compared with those of previously reported air filtration materials (Figure 4O). It was challenging to balance the specific surface area, filtration efficiency, pressure drop, circulating filtration efficiency, and quality factor. For example, the filtration efficiency of ZIF‐8@CNF@CF was high; however, its pressure drop (150 Pa) resulted in poor air permeability. Zr‐MOF@cotton showed a small pressure drop, but its filtration efficiency was only 36.9%. UiO‐66‐NH‐SO3H@cotton had a small specific surface area, and its filtration efficiency and pressure drop were intermediate. In contrast, ZIF‐67@BCFs had excellent filtering performance in all aspects, confirming their potential as an efficient and sustainable air filtration material. In addition, ZIF‐67@BCFs also showed the advantage of balancing air permeability and filtration efficiency when compared with some commercial materials,^[^ ^65^ ^]^ such as electrospun nanomaterials and natural fibers (Table S1, Supporting Information).

**FIGURE 5:**
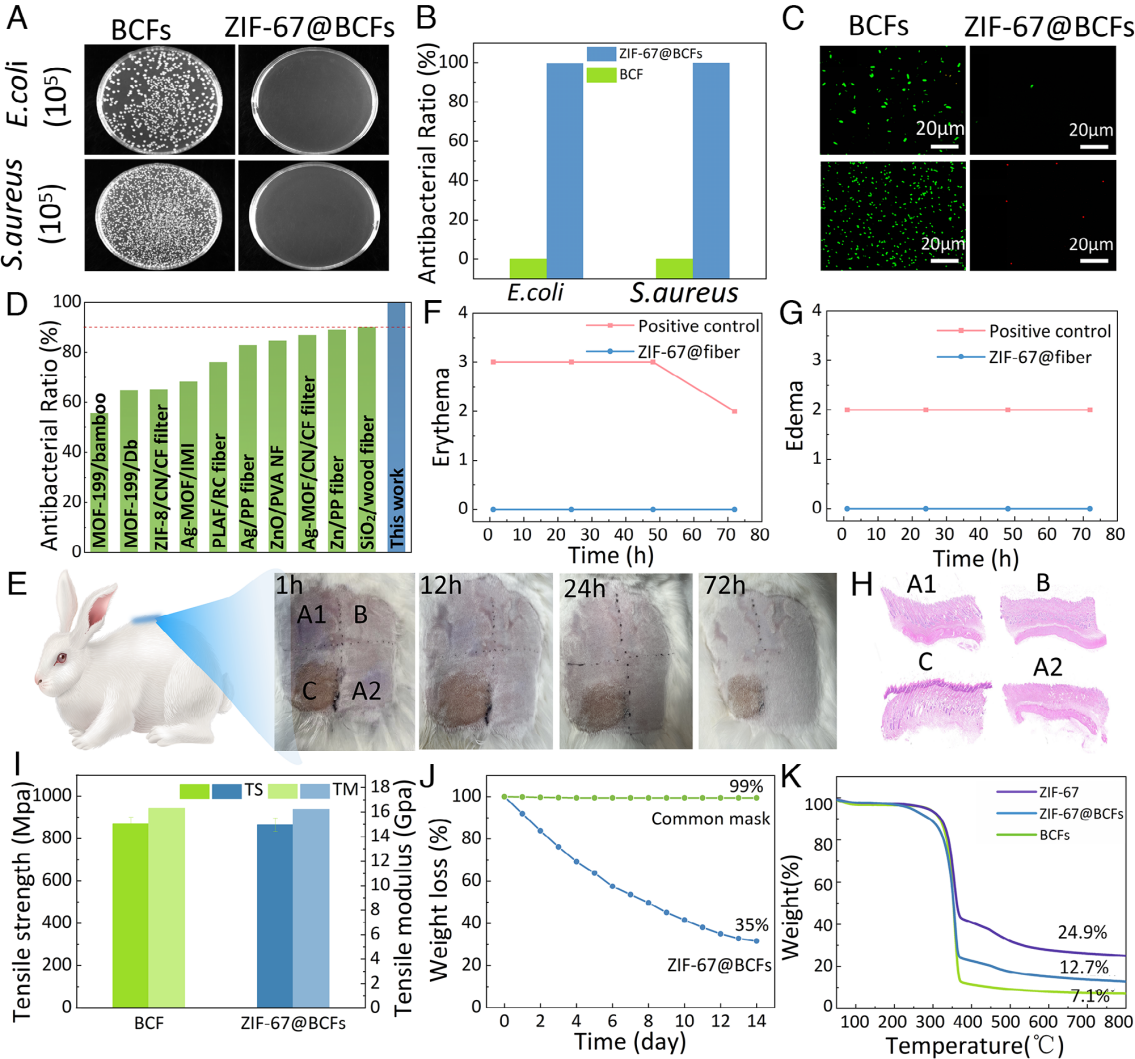
Plate count test showing the effects of BCFs and ZIF‐67@BCFs on the growth of *E. coli* and *S. aureus*, including (A) appearance of the counting plates, (B) antibacterial ratio, and (C) fluorescence microscope images. (D) Antibacterial ratios of ZIF‐67@BCFs and the other antibacterial materials. Scores of (E) erythema and (F) edema in skin irritation experiments after 72 h back stimulation (I) Mechanical strength of BCFs and ZIF‐67@BCFs. (G) Weight loss of commonly used masks and ZIF‐67@BCFs with α‐chymotrypsin. (K) TGA curves of ZIF‐67, BCFs, and ZIF‐67@BCFs.

The capability of ZIF‐67@BCFs in treating pathogenic air was studied by evaluating their antimicrobial efficacy using plate counting. The contact‐killing abilities of BCFs and ZIF‐67@BCFs against *E. coli* and *S. aureus* were tested. As shown in Figure 5A and Figure S7A, Supporting Information, the concentrations of *E. coli* and *S. aureus* in BCF were as high as 2.12 × 109 CFU mL^−1^ and 1.02 × 109 CFU mL^−1^, respectively. However, after incorporating ZIF‐67, they decreased to 2.1 × 106 and 6 × 105 CFU mL^−1^, respectively. The antibacterial rates against *E. coli* and *S. aureus* reached 99.8% and 99.9%, respectively (Figure 5B), indicating that ZIF‐67@BCFs were highly effective in inhibiting bacterial growth and blocking pathogenic agents, such as airborne bacteria. The antibacterial mechanism of ZIF‐67@BCFs could be attributed to the release of metal ions and organic ligands. The active metal ions in ZIF‐67 (Co^2+^) could bind to the hydroxyl groups of peptidoglycan membranes and oxidize membrane proteins and fatty acids, leading to cell membrane rupture and cell death. Additionally, the functional groups of the organic ligands in ZIF‐67 could also bind to intracellular Ca^2+^ and Mg^2+^, resulting in DNA breakage and cell membrane rupture.^[^ ^66^ ^]^ To verify the bacteriostatic effect, bacteria in contact with the BCFs and ZIF‐67@BCFs were washed and stained with propidium iodide and SYTO‐9 green fluorescence nucleic acid dye. As shown in Figure 5C, the bacteria in contact with the BCFs appeared green, suggesting that they could grow freely. Conversely, the bacteria in contact with ZIF‐67@BCFs appeared red, indicating growth inhibition and structural damage. Furthermore, the reusability of ZIF‐67@BCFs was assessed. As shown in Figure S7B–D, Supporting Information, no colonies were observed in the plate medium of ZIF‐67@BCFs, and the bacteriostatic rate maintained nearly 100.0% after washing one, three, and five cycles. Therefore, ZIF‐67@BCFs exhibited good reusability in terms of bacteriostatic rate, which was significantly superior to the recent reported materials (Figure 5D), such as MOF‐199/Bamboo (55.6%), MOF199/D‐Bamboo (64.9%), ZIF‐8@CNF@CF filter (65.2%), Ag‐MOFs/IMI (68.33%), Ag‐MOFs@CNF@CF filter (86.9%), Ag/PP fiber (82.8%), ZnO/PVA/NF (84.8%), and Zn/PP (89%).^[^ ^41^, ^42^, ^67^, ^68^, ^69^ ^]^

Considering that air filtration materials may contact the human body, the skin irritation experiments were performed on healthy white rabbits (weight >2.5 kg) following the ISO 10993.10‐2010 guidelines. The positive (C, PBS), negative (B, normal saline), and ZIF‐67@BCFs (A1 and A2) pairs were directly attached to the back skin of the rabbits (Figure 5E). The skin samples in the ZIF‐67@BCFs area showed no significant irritation within 1–72 h. The levels of edema and erythema were scored according to Table S2, Supporting Information. Edema and erythema in the positive control group were obvious, indicating that the rabbit skin had excellent sensitivity. Both the edema and erythema scores in the ZIF‐67@BCFs group were 0, indicating that the ZIF‐67@BCFs did not cause any irritation to the skin (Figure 5F,G). Simultaneously, the skin tissues were sliced and examined using hematoxylin and eosin (H&E) staining, and no apparent histopathological abnormalities were observed (Figure 5H). In addition, the skin irritation properties of moist ZIF‐67@BCFs were further verified. As shown in Figure S8, Supporting Information, the contact areas of ZIF‐67@BCFs (A1 and A2) produced no edema or erythema. In summary, these results indicate that ZIF‐67@BCFs possess excellent cytocompatibility, causing no skin irritation.

As a rich and sustainable resource, BCFs offer a strong skeleton with a porous and interconnected 3D network.^[^ ^51^ ^]^ The ZIF‐67 loading had an insignificant effect on the mechanical properties of the BCFs. The average tensile strength and elastic modulus of ZIF‐67@BCFs were 865.0 MPa and 16.1 GPa, respectively (Figure 5I), slightly lower than those of BCF (858.5 MPa and 16.2 GPa, respectively). In addition, biodegradation experiments using α‐chymotrypsin were performed on commonly used surgical masks and ZIF‐67@BCFs (Figure 5J).^[^ ^67^, ^70^, ^71^ ^]^ After 14 d, the degradation rate was approximately 1.0% for commonly used masks and 80.1% for ZIF‐67@BCFs. As shown in Figure S9, Supporting Information, the alkaline protease and lipase hydrolysis were 70.9% and 65.3%, respectively, for ZIF‐67@BCFs after 14 d, compared with approximately 1.0% for the common mask. These results demonstrate that ZIF‐67@BCFs are promising environmentally friendly degradable materials.

To assess the reusability of ZIF‐67@BCFs, their thermal stability and water‐washing resistance were investigated. Compared to the BCFs, the thermal stability of the ZIF‐67@BCFs analyzed by thermogravimetric analysis (TGA) was significantly improved (Figure 5K). The residual carbon at 800°C increased from 7.1% (BCFs) to 12.7% (ZIF‐67@BCFs), indicating a substantial enhancement in char formation in the presence of ZIF‐67. In addition, ZIF‐67@BCFs exhibited excellent water‐washing resistance with almost no mass loss after five continuous washes, probably because of the strong electrostatic interactions and hydrogen bonding between ZIF‐67 and the BCFs (Figure S10, Supporting Information). These results indicate that ZIF‐67@ BCFs possess excellent thermal stability and water‐washing resistance.

## 3 CONCLUSION

This study presents a bio‐inspired strategy to synthesize air filters via the synergistic combination of ZIF‐67 and BCFs. With the assistance of ultrasound technology, ZIF‐67 grew uniformly on the surface of porous BCFs, significantly increasing the specific surface area and strengthening the electrostatic binding between ZIF‐67 and BCFs. Therefore, the filtration efficiencies of ZIF‐67@BCFs for PM_2.5_ and PM_0.3_ could reach 99.3% and 98.6%, respectively, and its gas adsorption was 88.7% within 30 min. Furthermore, ZIF‐67@BCFs showed antibacterial properties (99.9% against *E. coli* and S. *aureus*), biocompatibility, biodegradability (80.1% degradation in 14 days), high mechanical strength, and good thermal stability, which are essential for their practical applications. More importantly, ZIF‐67@BCFs also demonstrated excellent reusability, and its PM_2.5_‐filtration efficiency remained at 98.4% even after five washes. Therefore, the hierarchical and multiscale porous materials hold significant promise in the realm of comprehensive air filtration.

## 4 MATERIALS AND METHODS

### 4.1 Materials

Moso bamboo (*Phyllostachys edulis*) aged 3−5 years was harvested in Zhejiang Province, China, and processed into strips after removing the green and yellow parts. The bamboo strips were then processed into thin slices with dimensions of 10 × 1 × 1 mm^3^ (length × width × height).

Sodium hydroxide (NaOH, 95%), sodium chlorite (NaClO_2_, 80%), cobalt nitrate hexahydrate (Co(NO_3_)_2_·6H_2_O, 99.99%), 2‐methylimidazole (C_4_H_6_N_2_, 98%), ethanol (CH_3_CH_2_OH, 99.8%) and methanol (CH_3_OH, 99.5%) were purchased from Aladdin. Phosphate buffer salt (PBS), tris (hydroxymethyl) amino methane (TRIS), lipase, alcalase, and α‐chymotrypsin were purchased from Macklin. Acetic acid glacial (CH_3_COOH, 99.5%) was purchased from Beijing Chemical Works. *E. coli* (ATCC 25922) and Staphylococcus aureus (*S. aureus*, ATCC 29213) were purchased from Feynman Biotechnology Technology Co., Ltd.

### 4.2 Preparation procedure for bamboo cellulose fiber (BCFs)

BCFs were prepared by a two‐step process to remove lignin and hemicellulose from bamboo slices. Initially, bamboo slices were immersed in a NaClO_2_ solution (1 wt.%, pH 4.5∼4.6) acidified with glacial acetic acid and heated to 80°C. The solution was changed to a fresh NaClO_2_ solution every 6 h until the bamboo completely turned white. Subsequently, the bamboo slices were rinsed with deionized water until a neutral pH to obtain delignified bamboo slices. Next, the delignified bamboo slices were immersed in a NaOH solution (2 wt.%) at 80°C. The solution was changed every 6 h until the slices turned white. After washing to a neutral pH, moist BCFs were obtained. Finally, supercritical CO_2_ was used to dry the BCFs.

### 4.3 Preparation procedure for ZIF‐67@BCFs

ZIF‐67@BCFs were synthesized by in situ growth of ZIF‐67 on BCFs using an ultrasonic method. First, BCFs were immersed in a Co(NO_3_)_2_·6H_2_O solution (1.3 wt.% in methanol) at room temperature for 12 h. Then, the same volume of 2‐methylimidazole solution (2.2 wt.% in methanol) was added in Co(NO_3_)_2_·6H_2_O solution, and the mixed solution was sonicated at 80% power for 4 h (KQ5200DE, Kunshan Ultrasonic Instrument Co., LTD). After that, the solution was filtered out with a filter screen, and the fiber was washed three times with water to remove the residual solution. Finally, the cleaned fibers were extracted and tiled in a round or square glass container for drying. Final dried ZIF‐67@BCFs were obtained after 24 h of drying at 80°C.

### 4.4 Characterization

The morphologies and structures of BCF and ZIF‐67@BCFs were characterized by SEM, EDX, TEM, AFM, and XRD. The chemical structures and surface functional groups were characterized by FT‐IR and XPS. Gas adsorption analysis (Micromeritics ASAP 2460, USA) was performed by a BET method using N_2_ as a carrier gas at 77 K. Before BET test, the sample was degassed under vacuum at 80°C for 10 h. The TGA curves were measured on an STA‐449F3 system (Netzsch) between 30 and 800°C at a heating rate of 10°C min^−1^ under N_2_ with a flow rate of 10 mL min^−1^. The tensile properties of the samples were tested on an in situ uniaxial microtension tester (Instron Microtester 5848, USA) at a loading rate of 0.005 mm s^−1^ following a previous study.^[^ ^32^ ^]^ Thirty replicates were used in each treatment group.

### 4.5 Degradable performance

Degradation experiments were performed in the solutions of lipase, alcalase (*Bacillus licheniformis*), and α‐chymotrypsin. Lipase (100 U mg^−1^, pH 8.0) and alcalase (200 U mg^−1^, pH 9.5) solutions were prepared in a TRIS buffer, and α‐chymotrypsin (800 u mg^−1^, pH = 7.4) was dissolved with a PBS buffer. The surgical mask filter layer (0.3 g) and ZIF‐67@BCFs (0.3 g) were placed in the three solutions at room temperature. Their masses were recorded for 14 consecutive days, and the mass loss was calculated to assess the degradation rate.

### 4.6 Antimicrobial activity analysis

*E. coli* and *S. aureus* suspensions were diluted in Luria‐Bertani (LB) liquid medium to 1 × 106 CFU mL^−1^, and 5 mL of the diluted solution was added to a sterile container. Then, 30 mg of BCFs and ZIF‐67@BCFs were added to the inoculated medium in an oscillator (37°C, 200 rpm) for 6 h. After the incubation, the bacterial liquid was continuously diluted 10 times with a sterile PBS solution, and 100 μL diluent was then evenly coated on the LB solid medium. The medium was incubated in a 37°C incubator for 24 h, and the colonies were recorded. Three replicates were used for each group, and the antimicrobial ratio was calculated as follows:

$$\begin{array}{ccc} \text{Antimicrobial ratio}\;(\%) & = & \left(1-\mathrm{CF}U_{\mathrm{modified}}/\mathrm{CF}U_{\mathrm{unmodified}}\right)\\ & & \times \;100\% \end{array}$$

where CFU modified is the colony value of a certain sample group (e. g. BCFs, ZIF‐67@BCFs), and CFU modified is the colony value of a blank control group.

### 4.7 Skin sensitization test

Skin sensitization test was performed on healthy 3‐month‐old female Japanese white rabbits according to the ISO 10993.10‐2010 standard. Japanese White Rabbit was purchased from Kelian Rabbit Industry Co., LTD., and its weight was greater than 2.5 kg. The ambient temperature of adaptive rearing was maintained between 20 and 26°C, humidity at 30–70%, and experiments were performed after 1 week of adaptive rearing. After shaving hair on the back of the rabbit (approximately 10 cm × 15 cm area), the BCFs, ZIF‐67@BCFs, positive control (sodium dodecyl sulfate, SDS), and negative control (saline) were directly attached to the skin. The mass of the BCFs and ZIF‐67@BCFs samples in the experimental group was all 0.5 g. Positive and negative controls were treated with 0.5 mL solvent soaked gauze. The material contact site was covered with a gauze block and fixed with a bandage for 6 h. After the contact period, the bandage was removed, and the corresponding plaque location was marked. Skin appearance was recorded after 1, 24, 48, and 72 h. The scoring system was shown in Table S2, Supporting Information, and erythema and edema scores were collected for each time period. The experiments were performed in triplicate, using one rabbit in each replicate. To study histopathological changes in skin tissue, skin sections after exposure were stained with H&E and observed microscopically.

### 4.8 Filtration capacity test

#### 4.8.1 Particulate matter filtration test

The filter testing for PM_10_ was performed using a customized instrument. PM_10_ was produced by a mixture of iron powder, nickel powder, and magnesium powder with 10 μm particle diameter and blown with a blower in a sealed bottle. The sample was placed in the module for PM_10_ interception with a testing area of 3.14 cm^2^ and an air volume of 2000 L min^−1^. A particle counter with a test particle size range of 0.3–10 μm was used to measure the number of particulates and further calculate the filtration efficiency (Videos S1 and S2, Supporting Information).

The PM_2.5_ and PM_0.3_ filtration performance of BCFs and ZIF‐67@BCFs were evaluated at room temperature using a TSI‐8130A filter tester (TSI Co., Ltd, USA). The samples and nonwoven fabric support were assembled to form a filter. The test area of the sample in the filter was 0.00785 m^2^. The oily and saline aerosol particulates with a diameter of 2.5 and 0.3 μm were produced by an atomizer, entered the test pipe, and reached the filter material. Pressure difference sensors are installed on both sides of the filter material, and the number of particles in the upstream and downstream particles of the filter material is monitored during the test. The whole test process was conducted at an air volume of 32 L min^−1^. The face velocity (*u*) through the filter is 6.8 cm s^−1^ according to the following formula:

$$u=Q/\left(F\times 36\right)$$

Where *Q* (m^3^ h^−1^) is the air volume, and *F* (m^2^) is the cross‐sectional area of the air filter.

In this work, *Q* = 1.92 m^3^ h^−1^, *F* = 0.00785 m^2^.

The measurement time and effective test area are 10 s and 113 cm^2^, respectively. The calculation formula for the filtering efficiency is shown as follows:

$$E=\left(1-\frac{C_{\mathrm{down}}}{C_{\mathrm{up}}}\right)\times 100\%$$

where E (%) is the PM filtration efficiency, *C*_down_ and *C*_up_ are downstream and upstream concentrations of particle, respectively.

The pressure drop in the filter was tested using a flow tester coupled with two electronic pressure sensors. Quality factor (*Q*_f_), a standardized parameter for comprehensively assessing the sample filtration performance, was calculated using the following formula:

$$Q_{f}=-\ln\left(1-E\right)/\Delta P$$

where *E* (%) is the PM filtration efficiency, and *P* (Pa) is the pressure drop.

#### 4.8.2 Formaldehyde adsorption test

The formaldehyde adsorption test was simulated using a customized 5 L glass vessel. The diluted formaldehyde solution was sprayed evenly, and its mass concentration in the closed space was measured in real‐time using a formaldehyde tester. The initial concentration of formaldehyde was set at 3 mg m^−3^. After the data were stable, the ZIF‐67@BCFs were placed in the glass vessel, and the concentration changes in formaldehyde and *T*_VOC_ were recorded.

#### 4.8.3 Incense cleaning test

An incense‐cleaning test was performed using custom‐made devices. The commercial incense (Sandalwood tower incense, Xiamen Jiayintang Culture and Art Co., LTD) was placed in a conical bottle to produce white smoke. BCFs and ZIF‐67@BCFs with an effective test area of 3.14 cm^2^ were placed at the junction of two glass bottles for interception. An airflow was applied to the incense bottle to accelerate the migration of pollutants, and the absorption and interception ability of BCFs and ZIF‐67@BCFs was measured.

## CONFLICT OF INTEREST STATEMENT

The authors declare no conflicts of interest.

## ETHICAL APPROVAL

All the animal experiments were complied with the guidelines of the Tianjin Medical Experimental Animal Care, and animal protocols were approved by the Institutional Animal Care and Use Committee of Yi Shengyuan Gene Technology (Tianjin) Co., Ltd. (protocol number YSY‐DWLL‐2023253).

## Supporting information

Supporting Information

Supporting Information

Supporting Information

Supporting Information

Supporting Information

## Data Availability

The raw data and processed data required to reproduce these findings are available from the author upon request.
