# Supplementary material for: Bio‐inspired hierarchical bamboo‐based air filters for efficient removal of particulate matter and toxic gases
Source: Exploration (Beijing). 2024 Jun 10;5(1):20240012. doi: 10.1002/EXP.20240012 (PMC11875449; doi:10.1002/EXP.20240012)
Supplement: Supplementary file 1 — Supporting Information [file EXP2-5-20240012-s004.docx]

Supporting Information

Bio-inspired Hierarchical Bamboo-based Air Filters for Efficient Removal of Particulate Matter and Toxic Gases

Qi Gao^1^, Jian Gan^2^, Pixiang Wang^4^, Yuxiang Huang^1*^, Daihui Zhang^2,3*^, Wenji Yu^1*^

1Research Institute of Wood Industry, Chinese Academy of Forestry, Beijing 100091, China

2Jiangsu Co-Innovation Center of Efficient Processing and Utilization of Forest Resources, Nanjing Forestry University, Nanjing 210037, China.

3Institute of Chemical Industry of Forest Products, Chinese Academy of Forestry, Nanjing 210042, China.

4 Center for Materials and Manufacturing Sciences, Department of Chemistry and Physics, Troy University, Troy, AL, 36082, USA

*Corresponding author.

E-mail: Y. Huang, yxhuang@caf.ac.cn; D. Zhang, dhzhang@icifp.cn; W. Yu, chinayuwj@126.com;


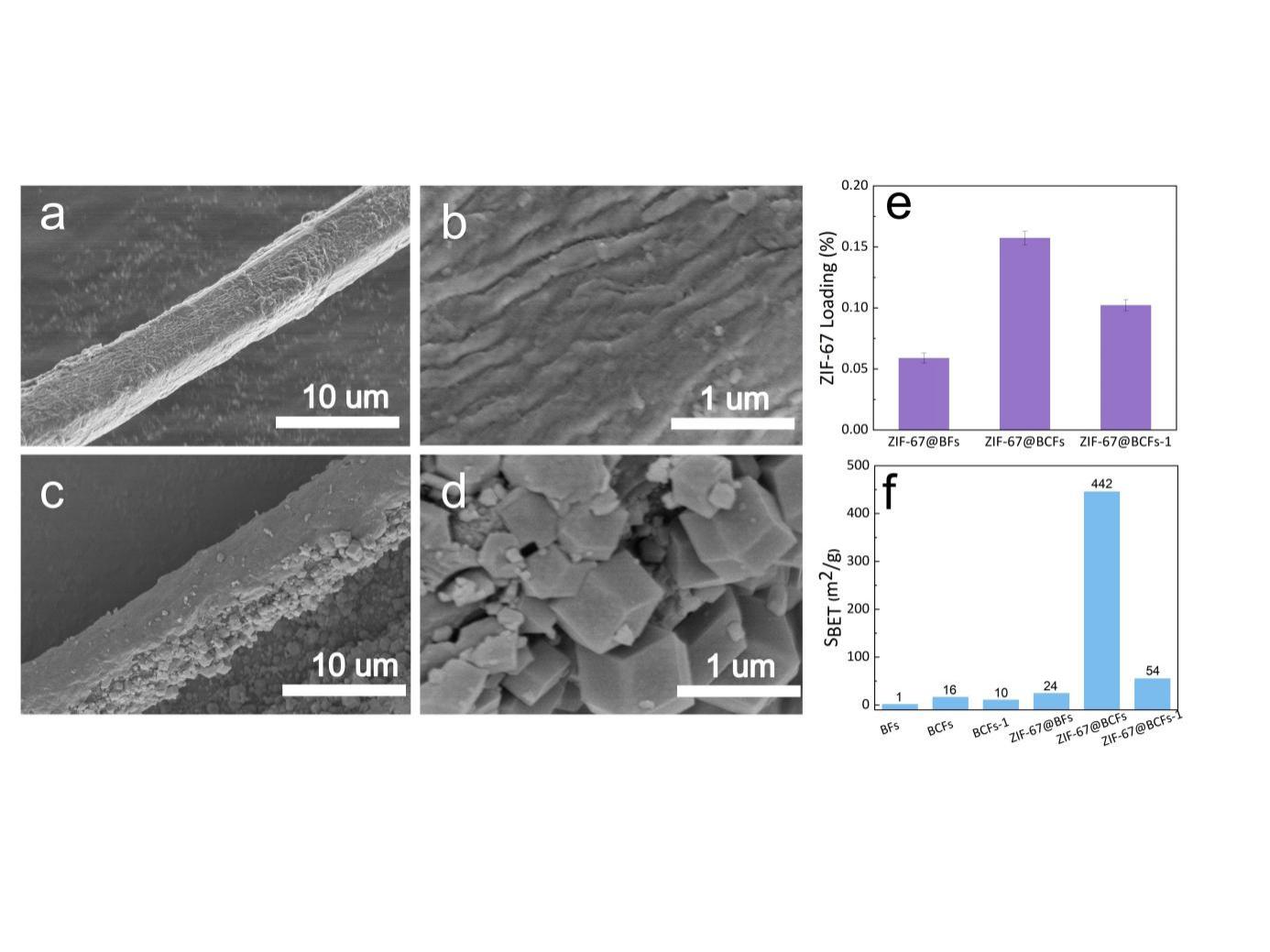


**Figure S1.** SEM images of (a-b) BFs and (c-d) ZIF-67@BFs. (e) ZIF-67 loading of ZIF-67@BFs, ZIF-67@BCFs and ZIF-67@BCFs-1. (f) The specific surface area of BFs, BCFs, BCFs-1, ZIF-67@BFs, ZIF-67@BCFs and ZIF-67@BCFs-1.


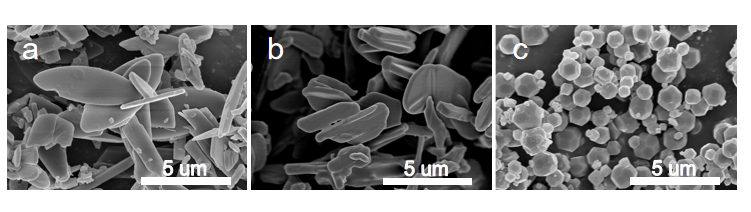
**Figure S2.** SEM images of ZIF-67@BCFs when the molar ratio of cobalt nitrate hexahydrate : 2-methylimidazole is (a) 1:8, (b) 1:15, (c) 1:30.


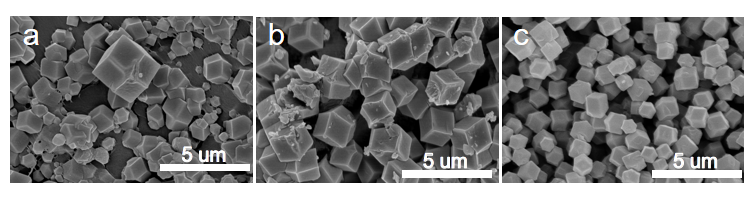


**Figure S3.** SEM images of ZIF-67@BCFs prepared separately at (a) room temperature, (b) 50℃, (c) ultrasound treatment.

In contrast, ZIF-67 crystals possessed numerous adsorption sites crucial for capturing and adsorbing gas molecules. More importantly, uninterrupted ultrasound for 4 h promoted a more uniform and dense growth of ZIF-67. In the current work, the ultrasonic worked continuously for 4 hours, and the temperature will rise by itself (Max to 50℃). Therefore, it is necessary to explore the influence of temperature in the ultrasonic process. At present work, the ultrasonic process is influenced by factors such as ultrasound temperature (Max to 50℃). In the preparation process of room temperature, 50℃ and with ultrasonic alone, the N2 adsorption capacity and SBET were not as substantial as with ultrasound technology (Fig. S4). In this case, ultrasonic alone was used to cool the water in the ultrasonic machine every half an hour, excluding the influence of temperature factors. The influence of ultrasound power on ZIF-67@BCFs was also explored, and the results showed that 80% ultrasound power can obtain the optimal N2 adsorption capacity and SBET (Fig. S4).


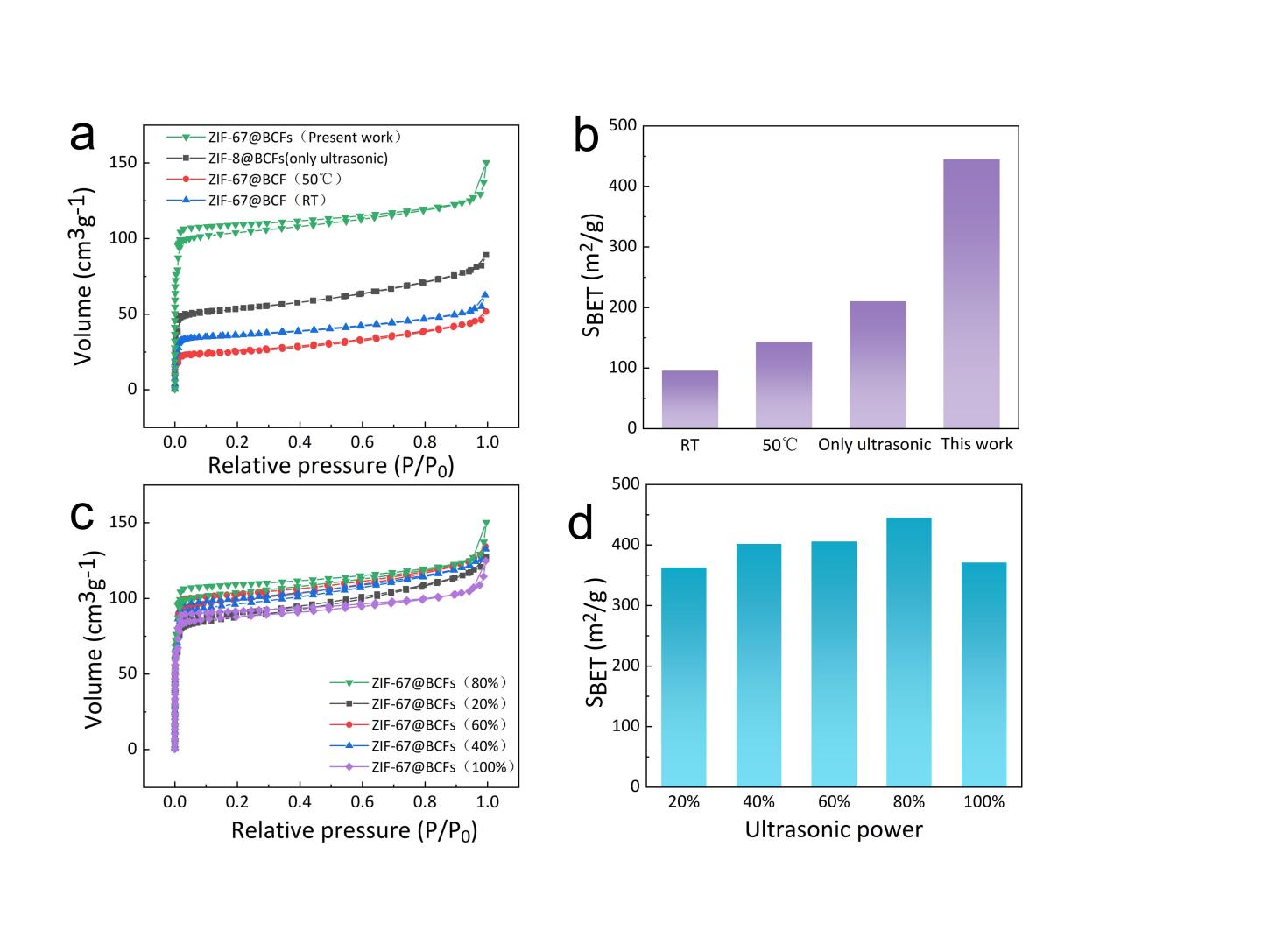


**Figure S4.** (a)The nitrogen adsorption isotherms and (b) specific surface area of ZIF-67@BCFs at room temperature, 50℃, only ultrasonic and present work. (c)The nitrogen adsorption isotherms and (d)specific surface area of ZIF-67@BCFs at ultrasonic power of

20%, 40%, 60%,80% 100%.


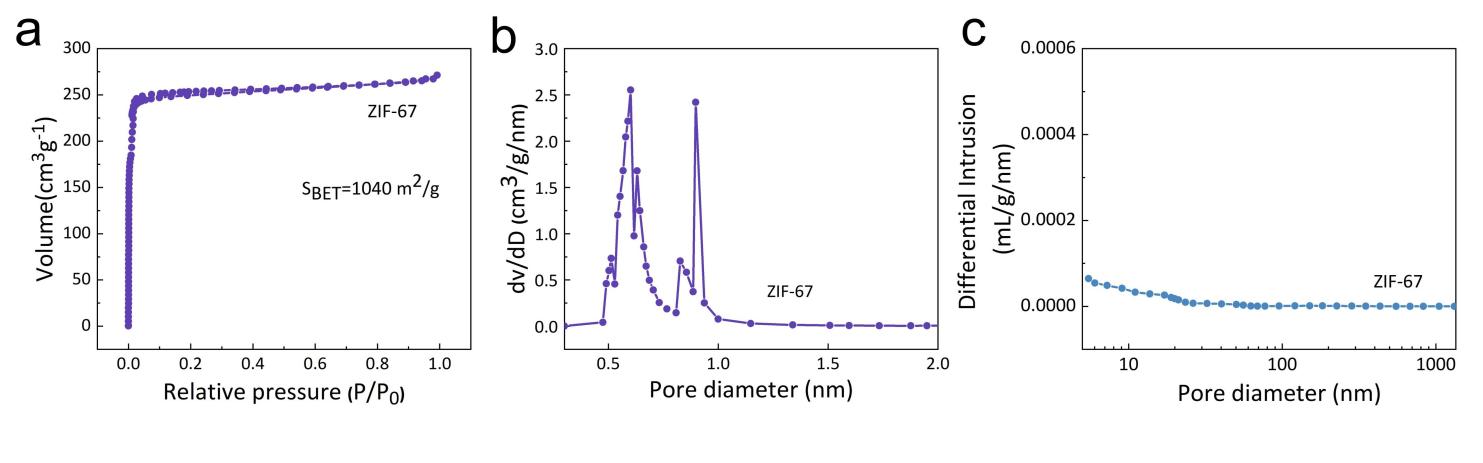


**Figure S5.** (a) N_2_ adsorption capacity and (b-c) pore distributions of ZIF-67.


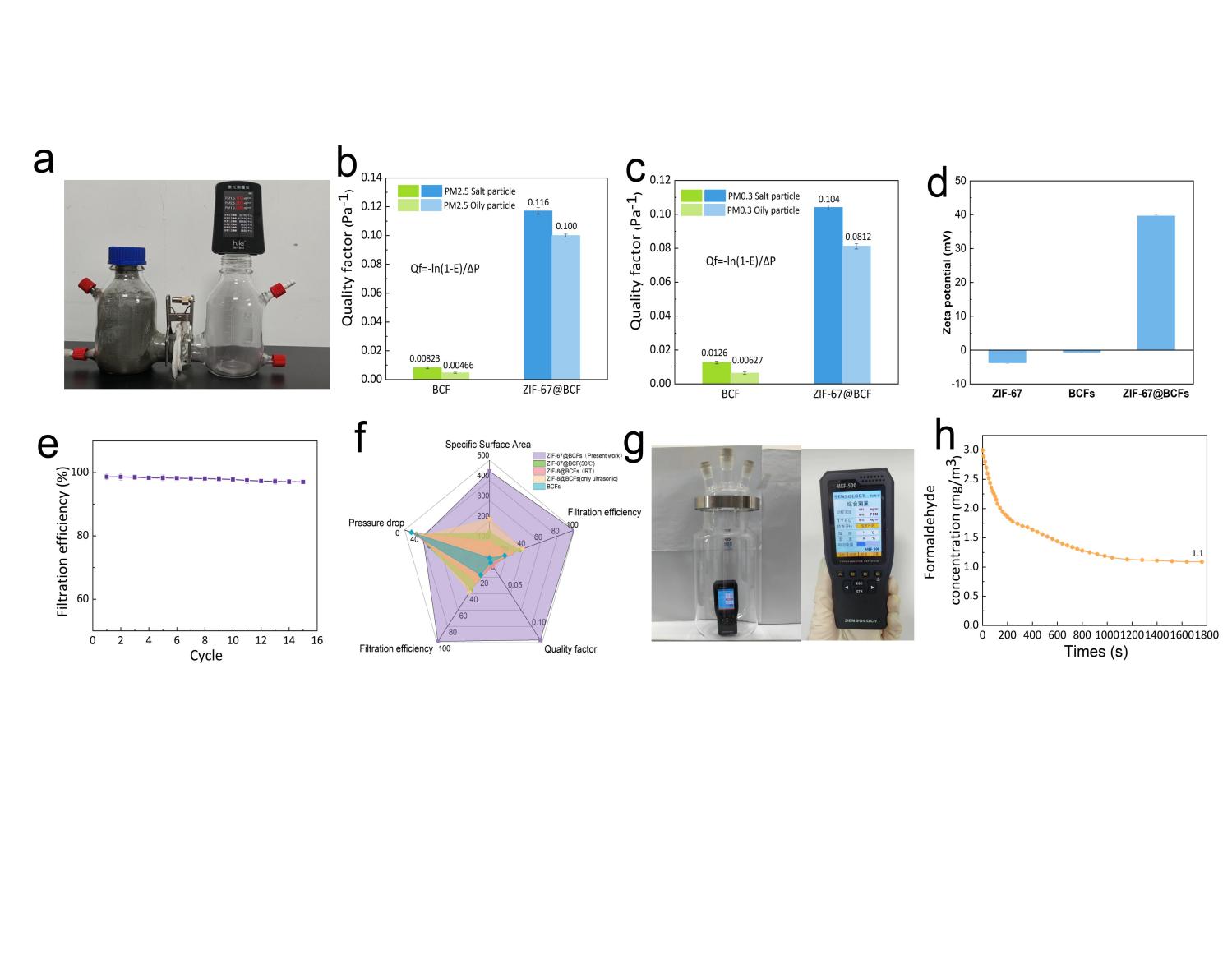


**Figure S6.** (a) Photo of the PM10 measuring device. (b) PM2.5 and (c) PM0.3 quality factor of BCFs and ZIF-67@BCFs. (d) Zeta potential of ZIF-67, BCFs and ZIF-67@BCFs. (e) PM0.3 filtration efficiency of ZIF-67@BCFs after washing. (f) Comprehensive comparison of the properties of ZIF-67@BCFs prepared by different processes. (g) Photo of the formaldehyde measuring device. (g) Adsorption properties of acticarbon for formaldehyde


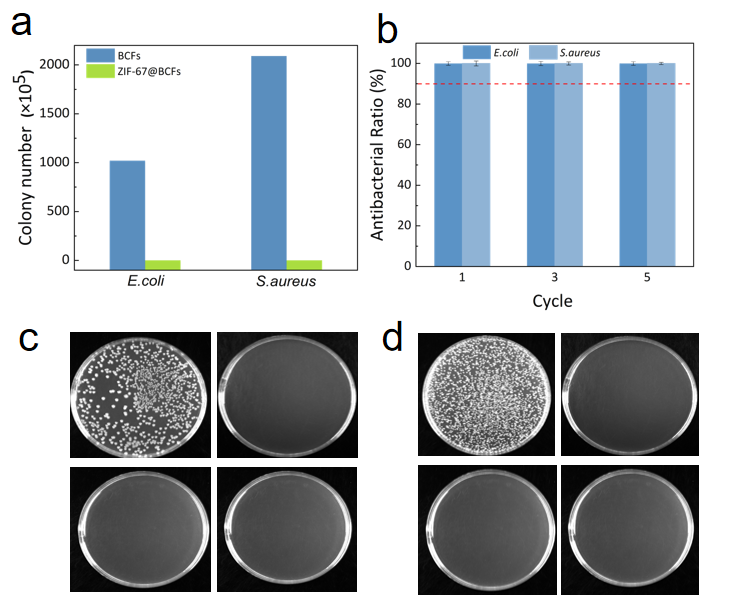


**Figure S7.** (a) Coloniy number of BCFs and ZIF-67 @ BCFs. (b)The antibacterial ratio and (c-d)counting-plates images and antibacterial rates of *E. coli* and *S**. aureus* after washing.


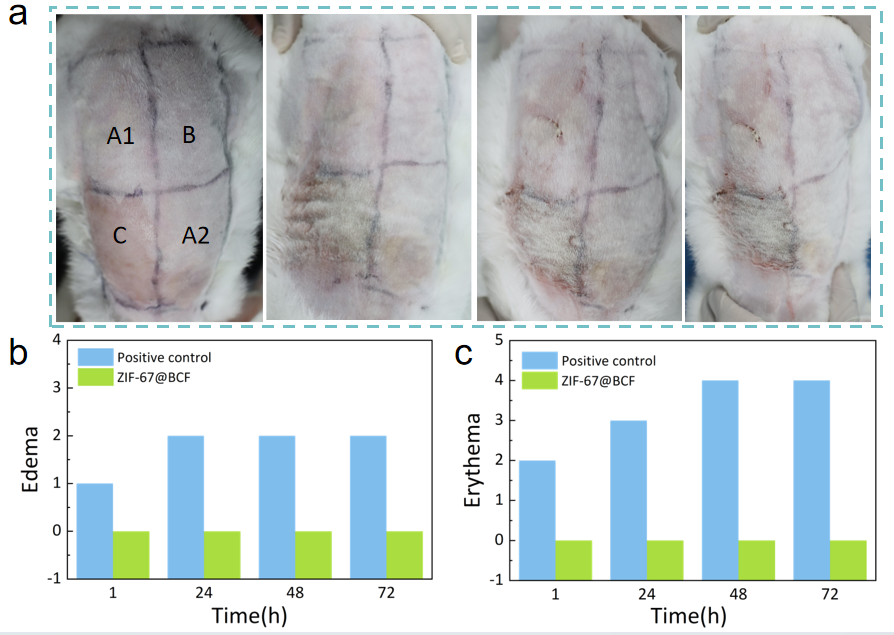


**Figure S8.** Skin irritation experiments of wet ZIF-67@BCFs in white rabbits at 1h, 24h, 48h,72h back (a) skin photos and stimulation scores of (b) edema and (c) erythema.


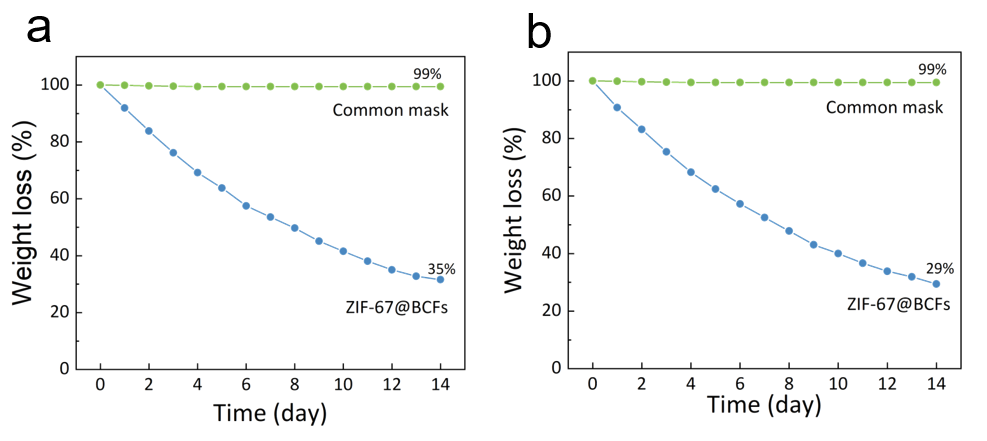


**Figure S9.** The weight loss of of (a) commonly used masks and (b) ZIF-67@BCFs with alkaline protease and lipase hydrolysis.


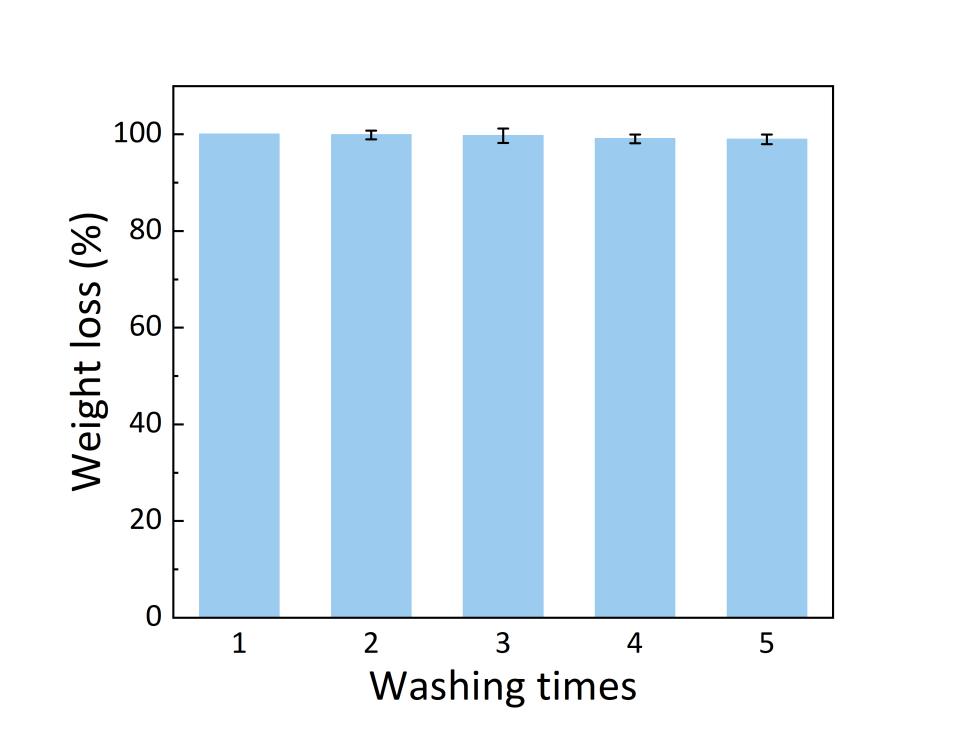


**Figure S10.** Weight loss of the ZIF-67 @ BCFs after washing.

**Table S1.** skin response scoring system


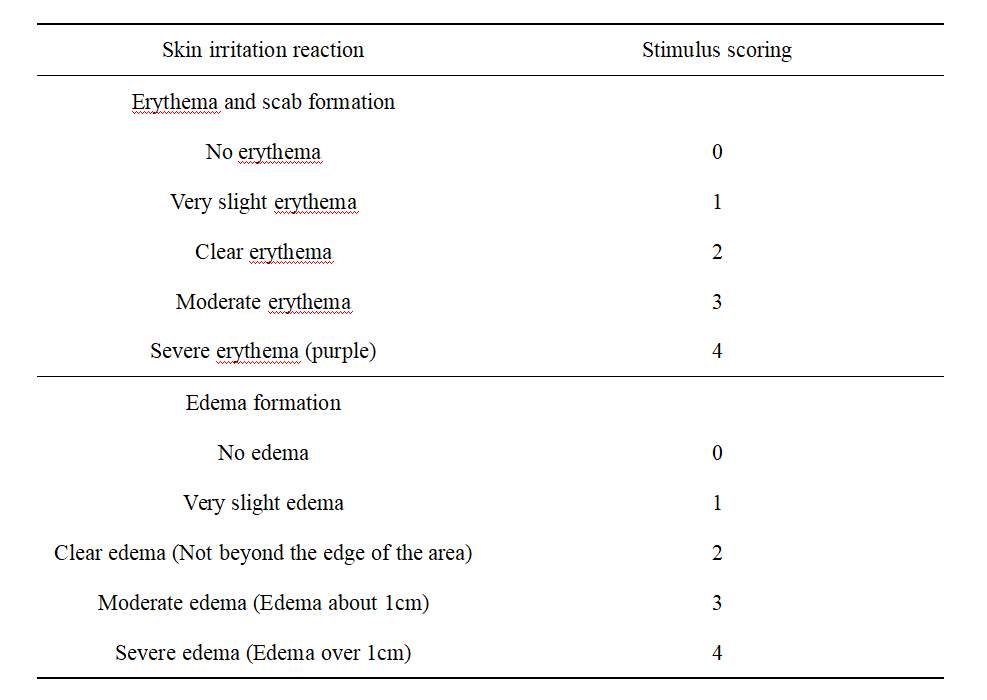


**Table S2.** Performance for the recently studied filters.

| Name in Ref. | PM type & Size(um) | Air resistance @ Face velocity | Efficiency | References |
| --- | --- | --- | --- | --- |
| ZIF-67@BCFs | Nacl 0.3um | 39 Pa @ 6.8cm/s (32L/min) | 98.6% | This work |
| PLA | KCl 0.3~0.5um | 40.8 Pa @ 5.3cm/s | 88.5% | ^[1]^ |
| PVDF | Nacl 0.3um | 27.8 Pa @ 5.3cm/s | 75.8% | ^[2]^ |
| PVDF | Nacl 0.3um | 52 Pa @ 5.3 cm/s | 97.4% | ^[3]^ |
| PVDF/SiO_2_ | Nacl 0.3um | 16 Pa @ 5.3 cm/s | 88.8% | ^[4]^ |
| PAN | Nacl 0.3um | 15 Pa @ 5.3 cm/s | 64.6% | ^[5]^ |
| PVDF/PTFE | Nacl 0.3~0.5um | 18 Pa @ 5.3 cm/s | 94.2% | ^[6]^ |
| PAN | Nacl 0.3~0.5um | 5.2 Pa @ 5.3 cm/s | 75.2% | ^[7]^ |
| PS/PAN | Nacl 0.3um | 54 Pa @ 5.3 cm/s | 99.9% | ^[8]^ |
| PAN/MOF | Nacl 0.3um | 60.7 Pa @ 5.3 cm/s | 90.2% | ^[9]^ |
| PAN/GO | Nacl 0.3um | 55 Pa @ 5.3 cm/s | 98.8% | ^[10]^ |
| PAN/PAA | Nacl 0.3~0.5um | 160 Pa @ 5.3 cm/s | 99.9% | ^[11]^ |
| PLA/PHB | Nacl 0.3um | 160 Pa @ 5.3 cm/s | 98.5% | ^[12]^ |
| PSA | Nacl 0.3um | 123 Pa @ 5.6 cm/s | 99.8% | ^[13]^ |
| PVB/Si_3_N_4_/FPU | Nacl 0.3~0.5um | 55 Pa @ 5.3 cm/s | 99.9% | ^[14]^ |
| PEI/SiO_2_ | Nacl 0.3um | 61 Pa @ 5.3 cm/s | 99.9% | ^[15]^ |
| PA | Nacl 0.3~0.5um | 46 Pa @ 5.3 cm/s | 99.3% | ^[16]^ |
| Worm silk | Nacl 0.3um | 98 Pa @ 5.3 cm/s | 96.2% | ^[17]^ |
| PAN/DMF | KCl 0.3~0.5um | 282 Pa @ 5 cm/s | 99.6% | ^[18]^ |
| PVA | PSL 0.3um | 80.6 Pa @ 5.3 cm/s | 96.8% | ^[19]^ |
| PVC/PA | Nacl 0.3um | 67.5 Pa @ 10cm/s | 98.7% | ^[20]^ |
| PVC | KCl 0.3 um | 23.1 Pa @ 5 cm/s | 58.6% | ^[21]^ |
| GO@PVDF NFMs | Nacl 0.3um | 28.17 Pa @ 5.3 cm/s | 95.19% | ^[2]^ |
| PU/Si3N4 | Burning incense PM_0.3_ | 25 @ 32 L/min | 79.36% | ^[22]^ |
| ZIF-67@BCFs | Nacl 2.5um | 43 Pa @ 6.8 cm/s | 99.3% | This work |
| PVDF/negativeions powder | Nacl PM_2.5_ | 80 Pa @ 5.3 cm/s | 99.9% | ^[23]^ |
| Worm silk | Ambient PM_2.5_ | 98 Pa @ 5.3 cm/s | 98.8% | ^[17]^ |
| PU | Burning cigarette PM_2.5_ | 10 Pa @ 4.1 cm/s | 98.9% | ^[24]^ |
| PAN/Ti_3_C_2_T_x_ | Burning incense PM_2.5_ | 42 Pa @ 5.3 cm/s | 99.7% | ^[25]^ |
| PVDF | Burning incense PM_2.5_ | 24 Pa @ 5.3 cm/s | 93.7 | ^[2]^ |
| PP electret filter | Nacl 1~3um | 68Pa @ 10cm/s | 63.5% | ^[26]^ |
| ZIF-8@PAN MOFilters | Al_2_O_3_ PM2.5 | 20 Pa @ 50 mL/min | 88.3% | ^[27]^ |
| UiO-67@Cotton filter | Burning incense PM_2.5_ | 42 Pa @ 20 cm/s | 73.3% | ^[28]^ |
| Ag-MOFs@ZIF-8@CNF@CF filter | Diethyl hexyl sebacate (DEHS) aerosol PM2.5 | 155Pa @5.3 cm/s | 95% | ^[29]^ |
| Electrical Ag NW/TiO_2_ NP-polyester fabric | Burning incense PM2.5 | 11Pa @ 5cm/s | 99% | ^[30]^ |
| Multilevel polarization-fields enhanced Schottky-junction nanofiber filter | Burning incense PM2.5 | 63Pa @ 20 L/min | 99.9% | ^[31]^ |
| GO@PVDF NFMs | Nacl  3 um | 28.17 Pa @ 5.3 cm/s | 95.19% | ^[2]^ |
| PVA | Burning incense PM2.5 | 175Pa @ 32 L/min | 70.81% | ^[32]^ |
| UiO-66-NH-SO3H/cotton | Burning incense PM2.5 | 38 Pa @ 20 cm/s | 79% | ^[33]^ |
| MiL-53(Al)/Aramid | Burning incense PM2.5 | - | 95.3% | ^[34]^ |
| Zr-MOF-NO2/cotton | Burning incense PM2.5 | 31 Pa @ 20 cm/s | 89.5% | ^[35]^ |
| PPC/ZIF-8 | Diethyl hexyl sebacate (DEHS) aerosol PM2.5 | 46 Pa @ 3.4 m^3^/h | 91.68% | ^[36]^ |
| ZIF-67 /CS composite gel | Diethyl hexyl sebacate (DEHS) aerosol PM2.5 | 197 Pa @ 32 L/min | 98.8% | ^[37]^ |
| ZIF-8/D-Wood | Burning incense PM2.5 | 37 Pa @ 50 cm/s | 89.8% | ^[38]^ |
| Cacl_2_/ MIL-101 /cotton | Burning incense PM2.5 | 30 Pa @ 20 cm/s | 92.1% | ^[39]^ |
| ZIF-8/PAN MOFilter | Burning incense PM2.5 | 20 Pa @ 50 mL/min | 88.3% | ^[27]^ |

**References**

| [1] | J. Zhang, G. Chen, G. S. Bhat, H. Azari, H. Pen, *J of Applied Polymer Sci* **2019**, *137*, DOI 10.1002/app.48309. |
| --- | --- |
| [2] | M. Chen, J. Jiang, S. Feng, Z.-X. Low, Z. Zhong, W. Xing, *J. Membr. Sci.* **2021**, *635*, 119463. |
| [3] | T. T. Bui, M. K. Shin, S. Y. Jee, D. X. Long, J. Hong, M.-G. Kim, *Colloid Surf. Physicochem. Eng. Asp.* **2022**, *640*, 128418. |
| [4] | X. Ding, Y. Li, Y. Si, X. Yin, J. Yu, B. Ding, *Compos. Commun.* **2019**, *13*, 57. |
| [5] | H. Liu, S. Zhang, L. Liu, J. Yu, B. Ding, *Adv. Funct. Mater.* **2020**, *30*, 1909554. |
| [6] | S. Wang, X. Zhao, X. Yin, J. Yu, B. Ding, *ACS Appl. Mater. Interfaces* **2016**, *8*, 23985. |
| [7] | Y. Yang, S. Zhang, X. Zhao, J. Yu, B. Ding, *Separation and Purification Technology* **2015**, *152*, 14. |
| [8] | R.-R. Cai, S.-Z. Li, L.-Z. Zhang, Y. Lei, *Sci. Total. Environ.* **2020**, *725*, 138297. |
| [9] | M. Lee, G. P. Ojha, H. J. Oh, T. Kim, H. Y. Kim, *J. Colloid Interface Sci.* **2020**, *578*, 155. |
| [10] | J. Li, D. Zhang, X. Jiang, X. Zhao, R. Hu, Y. Zhong, H. Zhu, *J. Materiomics* **2019**, *5*, 422. |
| [11] | Y. Liu, M. Park, B. Ding, J. Kim, M. El-Newehy, S. S. Al-Deyab, H.-Y. Kim, *Fibers Polym* **2015**, *16*, 629. |
| [12] | A. Nicosia, W. Gieparda, J. Foksowicz-Flaczyk, J. Walentowska, D. Wesołek, B. Vazquez, F. Prodi, F. Belosi, *Separation and Purification Technology* **2015**, *154*, 154. |
| [13] | Y. Li, J. Ming, D. Yuan, X. Ning, *Macro Materials &amp;amp; Eng* **2021**, *306*, DOI 10.1002/mame.202100081. |
| [14] | P. Jiang, X. Zhao, Y. Li, Y. Liao, T. Hua, X. Yin, J. Yu, B. Ding, *Compos. Commun.* **2017**, *6*, 34. |
| [15] | X. Li, N. Wang, G. Fan, J. Yu, J. Gao, G. Sun, B. Ding, *J. Colloid Interface Sci.* **2015**, *439*, 12. |
| [16] | B. Liu, S. Zhang, X. Wang, J. Yu, B. Ding, *J. Colloid Interface Sci.* **2015**, *457*, 203. |
| [17] | C. Wang, S. Wu, M. Jian, J. Xie, L. Xu, X. Yang, Q. Zheng, Y. Zhang, *Nano Res.* **2016**, *9*, 2590. |
| [18] | R. Al-Attabi, L. F. Dumée, J. A. Schütz, Y. Morsi, *Sci. Total. Environ.* **2018**, *625*, 706. |
| [19] | J. Matulevicius, L. Kliucininkas, T. Prasauskas, D. Buivydiene, D. Martuzevicius, *J. Aerosol Sci.* **2016**, *92*, 27. |
| [20] | Z. Shao, J. Jiang, X. Wang, W. Li, L. Fang, G. Zheng, *Nanomaterials. (Basel).* **2020**, *10*, 1706. |
| [21] | R.-R. Cai, L.-Z. Zhang, A.-B. Bao, *Building and Environment* **2018**, *131*, 210. |
| [22] | F. Liu, M. Li, W. Shao, W. Yue, B. Hu, K. Weng, Y. Chen, X. Liao, J. He, *J. Colloid Interface Sci.* **2019**, *557*, 318. |
| [23] | X. Zhao, Y. Li, T. Hua, P. Jiang, X. Yin, J. Yu, B. Ding, *ACS Appl. Mater. Interfaces* **2017**, *9*, 12054. |
| [24] | R. Chen, X. Zhang, P. Wang, K. Xie, J. Jian, Y. Zhang, J. Zhang, Y. Yuan, P. Na, M. Yi, J. Xu, *Nanotechnology* **2018**, *30*, 015703. |
| [25] | X. Gao, Z.-K. Li, J. Xue, Y. Qian, L.-Z. Zhang, J. Caro, H. Wang, *J. Membr. Sci.* **2019**, *586*, 162. |
| [26] | R. Thakur, D. Das, A. Das, *Journal of The Textile Institute* **2016**, *107*, 1456. |
| [27] | Y. Zhang, S. Yuan, X. Feng, H. Li, J. Zhou, B. Wang, *J. Am. Chem. Soc.* **2016**, *138*, 5785. |
| [28] | D. K. Yoo, H. C. Woo, S. H. Jhung, *ACS Appl. Mater. Interfaces* **2020**, *12*, 34423. |
| [29] | S. Ma, M. Zhang, J. Nie, J. Tan, B. Yang, S. Song, *Carbohydr. Polym.* **2019**, *203*, 415. |
| [30] | Q. Gao, J. Hu, Y. Huang, Q. Lin, W. Yu, *Ind. Crop. Prod.* **2022**, *188*, 115676. |
| [31] | P. Zhang, S. Zhang, D. Wan, P. Zhang, Z. Zhang, G. Shao, *J. Hazard. Mater.* **2020**, *395*, 122639. |
| [32] | H.-J. Kim, S. J. Park, D.-I. Kim, S. Lee, O. S. Kwon, I. K. Kim, *Sci Rep* **2019**, *9*, DOI 10.1038/s41598-019-43127-4. |
| [33] | D. K. Yoo, S. H. Jhung, *ACS Appl. Mater. Interfaces* **2019**, *11*, 47649. |
| [34] | K. Zhang, Q. Huo, Y.-Y. Zhou, H.-H. Wang, G.-P. Li, Y.-W. Wang, Y.-Y. Wang, *ACS Appl. Mater. Interfaces* **2019**, *11*, 17368. |
| [35] | H. C. Woo, D. K. Yoo, S. H. Jhung, *ACS Appl. Mater. Interfaces* **2020**, *12*, 28885. |
| [36] | T.-T. Li, X. Cen, H.-T. Ren, L. Wu, H.-K. Peng, W. Wang, B. Gao, C.-W. Lou, J.-H. Lin, *ACS Appl. Mater. Interfaces* **2020**, *12*, 8730. |
| [37] | J. Nie, H. Xie, M. Zhang, J. Liang, S. Nie, W. Han, *Carbohydr. Polym.* **2020**, *250*, 116955. |
| [38] | Z. Wang, F. Yin, X.-F. Zhang, T. Zheng, J. Yao, *Separation and Purification Technology* **2022**, *293*, 121095. |
| [39] | D. K. Yoo, H. C. Woo, S. H. Jhung, . **2021**, DOI 10.1021/acsami.1c02290. |
